# Designing a Transportation-Strategy Decision-Making Process for a Supply Chain: Case of a Pharmaceutical Supply Chain

**DOI:** 10.3390/ijerph18042096

**Published:** 2021-02-21

**Authors:** Afaf Haial, Loubna Benabbou, Abdelaziz Berrado

**Affiliations:** 1 Analysis, Modeling and Integration of Processes and Systems (AMIPS) Research Team, EMI School of Engineering, Mohammed V University in Rabat, Agdal 10090, Rabat, Morocco; berrado@emi.ac.ma; 2 Management Sciences Department, Lévis Campus, Université du Québec à Rimouski (UQAR), Lévis, QC G6V 0A6, Canada; Loubna_benabbou@uqar.ca

**Keywords:** transportation strategy, group decision-making process, multistakeholders, pharmaceutical supply chain, DELPHI method, group AHP method

## Abstract

Including an active participation of stakeholders along the transportation decision-making process is increasingly recognized as a necessary condition for reaching successful and high-quality decisions. This paper presents a framework for deciding on the appropriate transportation strategy for a supply chain from a multistakeholder perspective. It consists of three steps: (1) defining the transportation-strategy decision-making context and the objectives that must be achieved; (2) analyzing the actual transportation strategy regarding its three components: transportation network; transportation mode; and transportation insource/outsource, as well as identifying the stakeholders interested in the study; and (3) conducting a group decision making regarding each transportation strategy’s component, while involving the key stakeholders and taking into account the specificities of transported products. The proposed framework is then applied to a real case of the Moroccan public pharmaceutical supply chain, which has different features that distinguish it from other supply chains including its importance, urgency, and regulation. We employed the DELPHI method to determine the key stakeholders that should be involved in the decisional process. After that, we applied the group AHP method for selecting the appropriate transport-network design option while involving the identified key stakeholders.

## 1. Introduction

As one of the major logistical drivers, transportation has a large impact on both responsiveness and efficiency of the supply chain [1]. By ferrying goods from locations where they are sourced to locations where they are demanded, transportation provides the essential service of linking the whole supply chain from suppliers to customers [2].

In today’s constantly changing environment, knowing how to successfully navigate these changes and make the appropriate and effective transportation decisions to properly make the products arrive safe to customers at right time, right place, and right cost is a must [3]. With that in mind, transportation decisions in a supply chain can be characterized as strategic, planning, or operational, depending on the time period during which they apply [1]. The strategic decisions have a long-term impact lasting several years. Planning decisions cover a period of a few months to a year and include decisions such as production plans, subcontracting, and promotions over that period. Operational decisions span from minutes to days and include sequencing production and filling specific orders. In this paper, we are interested in the strategic decisions as long-term decisions that focus on the overall supply-chain transportation system.

Many authors [4,5,6] claim that continuous improvement and enhancement of transportation systems is required to satisfy increasing expectations of customers. In order to reach ever-increasing expectations, a solid and successful transportation decision-making process based on appropriate techniques and methods is highly desirable [7,8].

**Figure 1:**
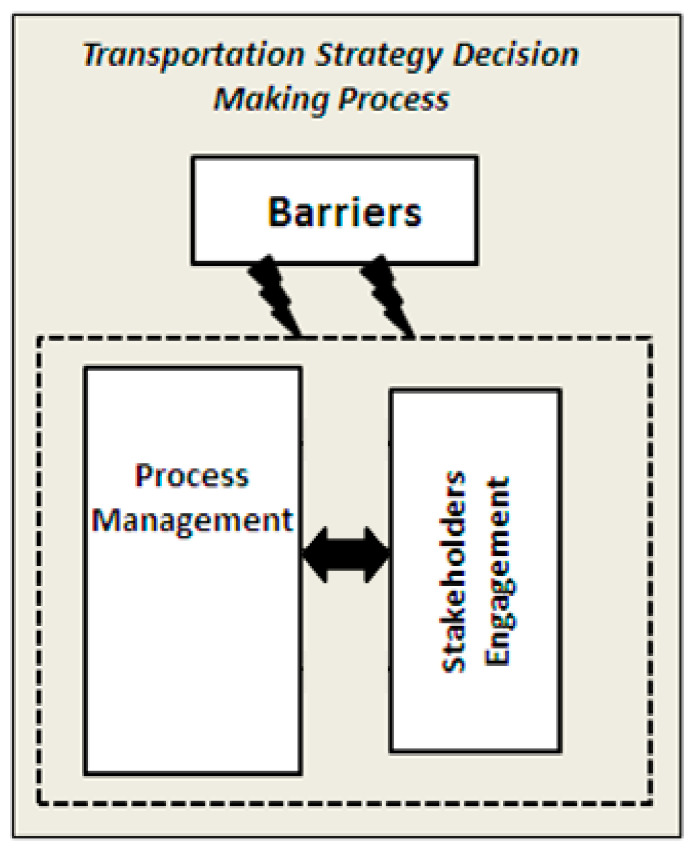
The components of the transportation decision-making process adopted from [5,9].

According to the literature [5,9], some of the main components that influence and facilitate the transportation decision-making process are as presented in Figure 1.

- Process management is concerned with the overall planning and coordination of the process. It structures the process into different stages. The theorists claim that the number of steps involved in the decision-making process varies depending on the nature of the problem [9]. In general, the most used and important steps in making transportation decisions can be characterized as a six-stage process. For each stage of the transportation decision-making process, the engagement one must be undertaken effectively [9].
  These stages are as follows:
  - Problem identification: The first stage of the process involves defining the problem to be addressed [10,11]. It includes the analysis of the current situation, the identification of objectives, and constraints, as well as the stakeholders to be involved.
  - Option generation: This stage involves the formulation of options and alternatives that look likely to achieve the objectives defined in the previous step. It includes inputs from key stakeholders [5,9].
  - Option assessment: This stage includes a technical analysis of each option to determine to what extent each option meets the stated objectives. In order to assess the different strategic alternatives, a multicriteria decision analysis (MCDA) can be applied. Ishizaka and Labib [12] claimed that the group-decision support methods (GDSM) are well suited to be applied as they are able to cope with the stakeholder-involvement concept.
  - Formal decision making: The decision is made by taking into account the views of stakeholder groups.
  - Implementation: This will include a process management plan that defines the detailed program of activities and schemes.
  - Monitoring and evaluation: This assesses the outputs and outcomes of the implementation against the process’s objectives.
- Stakeholder engagement covers a wide range of tools that can be used to ensure appropriate stakeholder involvement in all stages of the project decision-making process. Stakeholder’s participation in transportation decision-making process is emerging as a basic component for reaching successful and high-quality decisions [13,14].

The stakeholders are defined as any individual or group of individuals that can influence or are influenced by the decisions to be made [10]. Stakeholder engagement is considered to be the process of involving stakeholders’ concerns, needs and values in the decision-making process, in order to achieve more informed, transparent and successful decisions [5].

It has only been in more recent years that the stakeholders have become more engaged in making the appropriate transportation decisions and invited to give their ideas and express their needs and concerns [15]. The awareness of including the stakeholders in the transportation decision-making process is a consequence of the failure of many projects because of lack of consensus building [3]. Several researchers have pointed the importance of involving the stakeholders in the decision-making processes regarding different fields. In the transportation sector, Bickerstaff et al. [16] noted that stakeholder participation should be fitted into all stages of the transportation strategy making process. Also, Erkul et al. [17] discussed the benefits of involving the stakeholders and taking their views into consideration in transportation projects. In addition, Sussman et al. [18] highlighted the importance of stakeholder participation in all the phases of the transportation strategic process. Furthermore, Haial et al. [19] highlighted the importance of involving the concerns of different stakeholders in making the appropriate transportation decisions. Therefore, there has been a movement toward the development of transportation strategies in a more participative way.

- Barriers: A successful transportation process management recognizes the potential barriers that may restrict the scope of the project or hinder project completion. According to Kelly et al. [9], there are two broad categories of barriers that can be grouped for transportation. These include:
  (i) Contextual barriers which include the financial, legal, and institutional barriers.
    - Financial barriers: These are the barriers relating to the funding of a project.
    - Legal barriers: These concern the legal regulation and requirements that inhibit the implementation of decisions to be made.
    - Institutional barriers: These concern the rules and norms that stakeholders use to organize all forms of repetitive and structured interaction between them.
  (ii) Process barriers which can occur at any stage of the process, including:
    - Management barriers: These include any barriers relating to management of the decision-making process.
    - Communication barriers: These concern the problems associated with achieving consensus between stakeholders on possible courses of actions.

Therefore, transportation decision making is considered as a highly complex process, requiring the participation of multiple concerned stakeholders with different interests, expectations, and varying perceptions [2].

Several research works can be found in the literature that cover the topic of designing a transportation decision-making process. They are generally carried out to provide high-quality transportation decisions. Cascetta et al. [5] proposed a transportation decision-making process based on three parallel and intertwined processes: a cognitive rational approach to organizing the decision-making process, a five-level of stakeholder-engagement process, and a revised role of quantitative methods developed over the decades for the design and evaluation of transportation projects. In the proposed model, the decisions are still cognitive/rational, i.e., they are based on the comparison of alternative courses of action, with regard to their expected effects and objectives. Furthermore, decisions are made by exploring a limited number of alternatives until reaching a solution that satisfies decision makers and the majority of stakeholders.

In other work, Le Pira et al. [14] proposed a participatory transportation decision-making process that effectively includes the stakeholders’ opinions into the final decision. This process includes three main actors: planners and experts in charge of analyzing and modeling the transportation system by defining the plan structure for the final technical evaluations; stakeholders and citizens that are involved in all the planning phases for the definition of objectives, evaluation criteria, and alternatives; and decision makers in charge of the final decision supported by a performance-based ranking and a consensus-based ranking of plan alternatives.

Furthermore, Macharis et al. [11] proposed a framework for the evaluation of transportation-related strategic decisions. The proposed framework consists of seven steps. The first step concerns the definition of the problem and the identification of the alternatives. Next, the various relevant stakeholders, as well as their key objectives, are identified. Thirdly, these objectives are translated into criteria and then given a relative importance. Fourthly, for each criterion, one or more indicators are constructed. The fifth step concerns the evaluation of each alternative contribution to the objectives of all stakeholders. Next, the sixth step gives a ranking of the various alternatives and gives the strong and weak points of the proposed alternatives. The last step of the framework includes the implementation of the chosen alternative. Also, reference [6] proposed an agent-based model to support stakeholder involvement in deciding on the specific transportation policy.

In addition, reference [20] proposed a modeling approach to support stakeholder involvement in urban-freight-transportation policy making. The proposed approach consists of an integration of discrete choice models (DCM) with agent-based models (ABM) to account for stakeholders’ opinions in the policy-making process, while mimicking their interactions to find a shared policy solution. In addition, Ignaccolo et al. [21] presented a procedure for structuring a transportation decision-making problem while engaging the main stakeholders.

The analysis of these different transportation decision-making processes has shown the existence of many points of similarity. All of these processes involve a similar number of steps from the identification of the problem to be addressed, through the generation and assessment of alternatives to the implementation of the chosen alternatives and the monitoring of their impacts. Considering stakeholder participation and interaction in the process, all the previously described models have shown a vital interest in involving the stakeholders in all the phases of the process because of its benefits in making successful decisions. They considered that interaction is fundamental to the success of the decision-making process, because it allows one to increase the degree of consensus of the collective decision.

However, we have noticed that these studies do not deal with determining decisions regarding the transportation in a supply chain. We therefore determine a lack in establishing a decision-making process that helps in deciding on strategic transportation decisions in a supply chain.

Moreover, the transportation of pharmaceutical products, in particular, represents one of the most complicated operations in the pharmaceutical supply chain because of the sensitive nature and the specific characteristics of pharmaceuticals that require a high degree of attention during transportation [22]. Improper transportation of pharmaceuticals not only affects customer satisfaction and supply-chain profit but could also present a real threat to public health and safety [22]. Thus, making the appropriate pharmaceutical transportation decisions is a complex and challenging task. Several research studies have been conducted concerning the transportation of pharmaceutical products. Reference [23] proposed a risk-assessment framework to select shipping lanes for pharmaceutical products. Kumar and Jha [24] proposed a new application of principles of supply-chain management to the pharmaceutical-goods transportation practices in order to make quality products arrive to customers in a timely manner across the globe. Moreover, the analysis of the literature has shown that the majority of the studies used mathematical modeling to deal with transportation problems. For example, Goodarzian et al. [25] proposed a mathematical programming with the objectives of minimizing the total costs and the delivery time of pharmaceutical products and of maximizing the reliability of the transportation system. In addition, Izadi and Mohammad Kimiagari [26] proposed a mathematical model to design a distribution network under demand uncertainty for a pharmaceutical distribution companies in Iran. Therefore, we have noticed a gap in existing literature related to the design of transportation systems, while using technical methods other than mathematical modeling like the multicriteria decision-making methods.

In this paper, we are interested in presenting a participatory framework for deciding on the appropriate transportation strategy for a pharmaceutical supply chain while involving the interested stakeholders.

The rest of this paper is organized as follows: The suggested framework is presented in Section 2. Section 3 consists of a discussion regarding the key components of the proposed transportation strategy decision-making process. An illustration of the proposed framework in designing the appropriate transportation strategy for the Moroccan public pharmaceutical supply chain is presented in Section 4. Finally, the results are discussed in Section 5.

## 2. Suggested Framework for Designing Appropriate Transportation Strategy for a Supply Chain

Developing a successful supply-chain transportation strategy involves decision making regarding several components. Choosing the appropriate transportation mode, designing the transportation network, and deciding which part of the transportation process will be outsourced and which one will be insourced are the major decisions that must be made, while involving the relevant stakeholders and taking into account the specificities of products to be transported, in order to design an effective transportation strategy.

**Figure 2:**
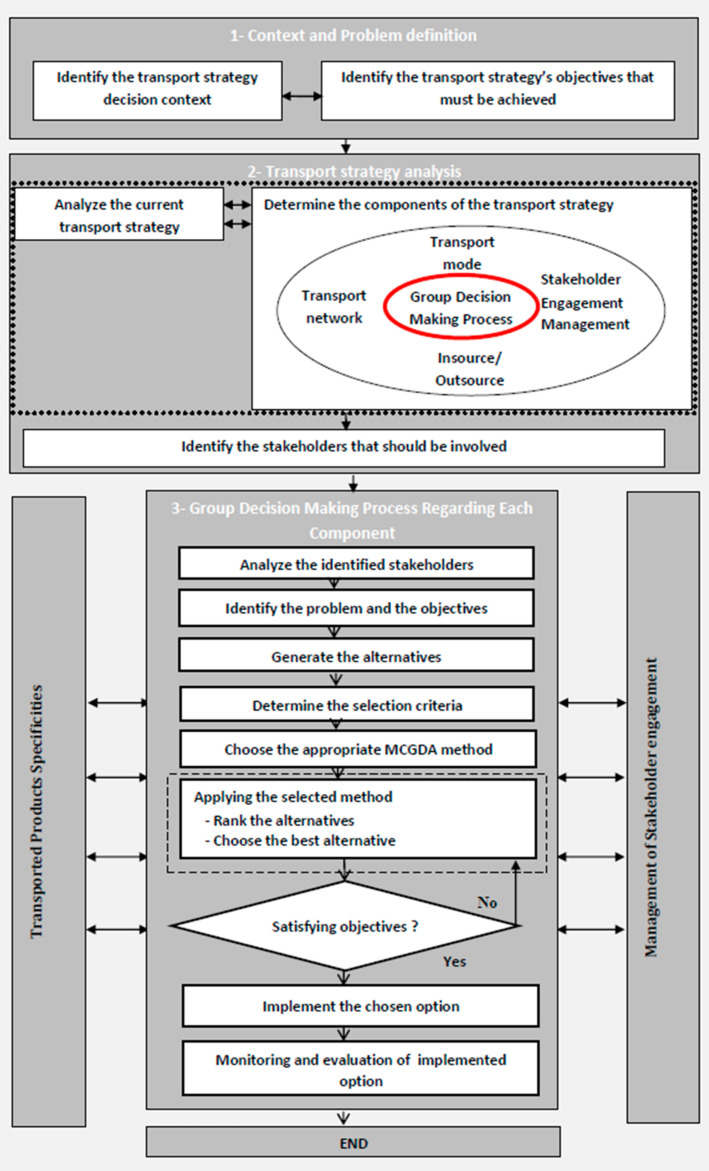
Framework for designing a transportation strategy for a supply chain.

Based on the literature review, we proposed a transportation strategy decision-making process that effectively involves the stakeholders in all the phases of the process in order to help the decision makers in making the appropriate and the most shared decisions. Figure 2 schematizes the proposed framework. Defining the transportation strategy decision-making context and the objectives that must be achieved is the first step of the framework. The second step consists of analyzing the actual transportation strategy regarding its three components: transportation network, transportation mode, and transportation insource/outsource, as well as identifying the stakeholders interested in the study. The third step of the proposed framework refers to the group decision making regarding the three components of the transportation strategy that have already been mentioned, while involving the stakeholders and taking into account the specificities of transported products. Many participatory techniques can be applied to involve the stakeholders in the decisional process, such as newsletters, reports, presentations, public hearings, interviews, questionnaires, surveys, workshops, participatory mapping, focus groups, newsletters, meetings, and informal discussions. The third step requires high input from stakeholders and their support for the decisions to be made. Deciding on the priority of these decisions, i.e., which decision will be made first depends on the stakeholders’ needs and preferences.

The group decision-making process involves several steps; the first one is analyzing the identified stakeholders to retain the most critical ones to be fully engaged in the decision process in order to satisfy their needs and expectations. The second step consists of identifying the problem associated with each component and the objectives that must be achieved in order to reach the final goals related to the transportation strategy. The third step involves the generation of the alternative options, the determination of the different criteria, and then a multicriteria group decision analysis (MCGDA) method is applied in order to determine the best option that meets the stated objectives, while taking into account the views of stakeholder groups. In fact, the reason behind the choice of the multicriteria group decision-making analysis method to be applied for deciding on transportation strategy is due to the presence of different stakeholders with multiple conflicting criteria involved in the decision-making process.

The fourth step of the proposed framework includes all necessary preparatory resources needed in order to bring the selected option to the point of operation; this stage can be supported by the participation of the stakeholders. Finally, an evaluation of the outputs of the implemented alternative is undertaken in order to determine whether the objectives have been met.

In the following section, we present a literature review regarding the key components of the proposed transportation strategy decision-making process, with the intent of creating a clear understanding of the problem at hand, as well as analyzing and defining the relevant criteria used in the literature in evaluating the problems associated with these components.

## 3. Key Components of the Proposed Transportation Strategy Decision-Making Process

In this section, we briefly review literature devoted to the key components of the suggested transportation strategy decision-making process: namely, the design of the transportation network, the choice of the appropriate transportation mode, deciding on transportation outsourcing/insourcing, products portfolio analysis, and management of stakeholder engagement. We also define the relevant criteria used in the literature in evaluating the problems associated with these components.

### 3.1. Transportation Mode

The movement of products from a source to a destination can be undertaken using one or a combination of the following modes of transport: air, package carriers, truck, rail, water, and pipeline [1]. Each of these modes has different characteristics, and any of them can be considered the best under different circumstances, depending on different criteria [27].

Rail or railway transportation is the cheapest, quickest, and best suited for carrying heavy, bulky, and not very time-sensitive goods over long distances. Some of the advantages are constancy, low-cost guarantee, and greater reliability, and they are not affected by the weather and traffic conditions. The main disadvantages are inflexibility and specified routes between fixed terminals; moreover, they do not stop at intermediate points [28].

Sea transportation is suited for carrying very large and bulky loads at low cost. However, the damage risk is high, transit times are long, and there is a limitation and inflexibility with regard to finding appropriate ports.

In addition, air transportation is the most convenient mode, when a slow speed is unacceptable. It is, however, the most expensive mode [1].

Of all the transportation modes available to use in the movement of products, trucking, is still the single most widely used mode [29]. A variety of light, medium, heavy, and very heavy trucks exist today with a diversified range of trailers, in order to satisfy almost any products transportation demand [30]. From an operational point of view, trucking services are classified in the two following modes: TL (truckload) and LTL (less-than-truckload) [1].

Full truckload shipping is described as a transportation chain with only one link, where source and destination are peer-to-peer connected through a direct, noncombined transportation [31], so that no additional handling or sorting costs occur [32]. Typically, truckload is suited for large shipping even between manufacturing facilities and warehouses or between suppliers and manufacturers. It is also a considerably faster mode to transport products by not having the driver stop for multiple pickups or having to load and unload freight throughout the trip [27].

On the other hand, the term LTL is appropriate when transporting shipments in small lots that do not fill an entire truck [30]. Less-than-truckload shipments take longer than truckload shipments, because of other loads that need to be picked up and dropped off [1]. It is the most cost-effective way to transport products because of the high degree of consolidation that carriers can achieve for the loads carried [1]. The complexity of the LTL industry is about the location of consolidation centers, assigning of loads to trucks, and scheduling and routing of pickup and delivery [1].

Furthermore, in order to take advantage of the benefits offered by various transportation modes and compensate for deficiencies, shippers often use more than one mode to transport products. The intermodal transportation, as this integrated approach is called, has become increasingly common and applicable. It provides a flexible response to the changing supply-chain management requirements in global markets and distribution systems [32].

In the selection of a suitable mode from the different modes previously described to use for each part of the transportation network, all the characteristics related to the concerned mode of transportation have to be considered. Under certain conditions, the choice of the suitable transportation mode may seem obvious, but a comparison that depends on a variety of criteria may be nevertheless required. As shown in Table 1, several researchers have studied the determination of the suitable transportation mode under the consideration of a variety of criteria. These include:

- Customers/suppliers density and distance (C1): This criterion should also be considered when deciding on the degree of temporal aggregation to use when supplying customers. Moreover, the selection of the mode to ship goods depends on the accessibility and the geographic location of the customers/suppliers.
- Shipment size (C2): Decision makers should take into consideration the weight, density, and shape of the products as well as its packaging in choosing the appropriate transportation mode. For instance, lightweight products can be transported by land or air, while heavier products are suited for railroads or waterways.
- Product characteristics (C3): This criterion is involved with product-related features:
  - Requirements associated with the type of the freight to ship, such as goods for fast consuming (highly perishable) or frozen products.
  - Financial value of the transported freight.
- Delivery time (C4): Duration of the overall transportation process.
- Total cost (C5): This contains all cost factors that are involved in the transportation process.
  - Transportation cost for shipment of products from source to destination.
  - Cost of damages to freight incurred at the transportation or transshipment stages.
  - Information system cost to provide coordinated transportation.
- Flexibility (C6): This criterion handles the unexpected changes in demand during the transportation, capacity flexibility, and routes flexibility.
  - The ability to meet the unexpected changes in demand.
  - The ability to change the volume and weight capacity of the transportation vehicles.
  - The ability to change the transportation route at any time during transportation.
- Safety criteria (C7): This involves the safety problems related to the transportation process.
  - Probability of avoiding damage and loss of quality of goods.
  - The accidental rate in a determined time period.

### 3.2. Transportation Outsourcing/Insourcing

In today’s constantly changing market, managing transportation is a key factor for a more efficient and cohesive supply chain. Deciding which part of the transportation process will be outsourced and which one will be insourced is one of the most important strategic decisions that have a large impact on the total supply-chain profit [1].

In fact, transportation managers should decide whether to outsource the entire transportation process or to outsource a part of it, and they should also determine the level and extent of outsourcing of each part based on the growth in total supply-chain profitability [1]. Outsourcing is the delegation of one or more business processes or functions to an external provider who, in turn, owns administers and manages the selected process or functions base upon defined and measurable performance metrics [44].

Recently, outsourcing has become the most preferred strategic option for the most important structures in order to keep focus on their core businesses [45]. However, there are a large number of structures that choose not to outsource and maintain control of their supply chains internally or in-house [46]. Thus, the decision of insourcing/outsourcing represents one of the more complex choices facing the transportation managers.

Choosing either to outsource or to insource is a complex decision-making process, arising out of a variety of several factors. Many authors present different factors critical to deciding between outsourcing and insourcing. A deep analysis of the literature devoted to the insourcing/outsourcing decision-making process sorted different criteria that are mainly used by the decision makers in making the right insourcing/outsourcing decisions (See Table 2). These include:

- Total cost (C1): Estimating the cost of outsourcing the transportation or keeping it in-house is an important criterion to be taken into account while making the decision. A deep cost–benefit analysis must be done in order to make the right decisions.
- Core competency (C2): Outsourcing is a very helpful tool which enables the company to concentrate on its own core competencies and to increase its efficiency and profits.
- Technological capability (C3): Outsourcing can offer the possibility to benefit from the new technologies that meet the changing market conditions, and also to access the latest technology and external skills and talent.
- Flexibility (C4): Outsourcing allows organizations to create lean supply chains that can quickly change to meet changing requirements, demands, or technologies.
- Responsiveness (C5): Outsourcing improves the responsiveness of the supply chain.
- Quality (C6): Outsourcing offers an improvement of the quality, in the sense of offering a specific level of technological capability that is required to meet the minimum requirements for the product quality.
- Timeliness (C7): The outsourcing decreases the delivery time when the customer orders are small in the sense of aggregating different firm’s orders.
- Information security (C8): The outsourcing can lead to the failure of the product traceability and the leakage of the information.
- Quality Control (C9): Insourcing offers the advantage of a high degree of control and visibility over all the operations.
- Product complexity and specificity (C10): In making the insourcing/outsourcing decisions, it is necessary to take into account the nature of product to ship in order to avoid damage.
- Risks (C11): Risks related to outsourcing, such as risk of strategic information leakage and risk of the product leakage
- Shipment size (C12): Outsourcing is a better option when shipment sizes are small, whereas owning the transportation fleet is better when shipment sizes are large.

A summary of the reviewed studies on the outsourcing/insourcing decision-making process under considering the different criteria that have been already discussed is shown in Table 2.

Moreover, the involvement of the public and the stakeholders is another important dimension that must be taken into account in making the insourcing/outsourcing decisions.

### 3.3. Transportation Network

The design of a transportation network affects the performance of the whole supply chain by implementing the infrastructure in which several transportation decisions are made [1]. It used to find the way of transporting products from several sources to several destinations, so that the total cost can be minimized without sacrificing customer responsiveness [1].

There are a variety of transportation-network design options [1,63].

**Figure 3:**
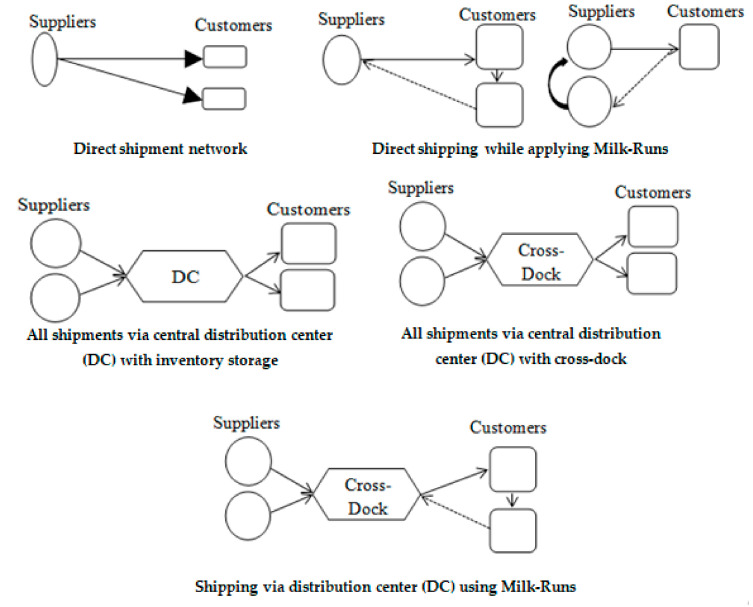
The different transportation-network design options according to [1].

They include the six options given below (See Figure 3):

(a) Direct-shipment network: The products are transported directly from suppliers to customers.
(b) Direct shipping while applying milk runs: The goods are either transported directly from one supplier to multiple customers or from many suppliers to one customer.
(c) All shipments via central distribution center (DC) with inventory storage: Products are shipped to customers via a distribution center which is built for each region of the country. The products are held in inventory at the distribution center.
(d) All shipments via central distribution center (DC) with crossdock: Products arrive from many suppliers into inbound trucks to be transformed into smaller shipments that are then loaded onto trucks going to each customer.
(e) Shipping via distribution center (DC) using milk runs: Products are shipped from suppliers to distribution centers to be then transported on milk-run to each customer by consolidating small shipments.
(f) Tailored network: Combination of different transportation-network options based on customer and product characteristics.

Choosing the appropriate transportation-network design option is one of the most important operations that transportation managers should focus on. In this sense, we have proposed in a previous work [63] a framework for designing an appropriate transportation network for a given supply chain.

An in-depth analysis of the literature has allowed us to draw up a list of a variety of criteria that have been mainly used in designing transportation networks. These include:

- Delivery time (C1): The actual time between placing an order, and receiving the delivered product;
- Number of trucks (C2): The use of milk run reduces the number of trucks utilized for shipping pharmaceutical products;
- Transportation cost (C3): Consolidating shipments lowers transportation cost;
- Information system cost (C4): The use of milk runs increases the cost of an information system, because it requires a significant degree of coordination;
- Size of served area (C5): The design of the appropriate transportation network must be suited to the size of the area to be served;
- Transportation risk (C6): Risk is the probability of harm or damage occurring from exposure to a hazard, and the likely consequences of that harm or damage. It may refer to the concept that the transportation plan is not reliable in practice. It also refers to the potential impact on environment and human, for example, when it comes to the transportation of hazardous materials. Transportation risk reduction is one of the major interests of the public and governments around the globe [64]. Therefore, the selected transportation-network option should take into account the minimization of risk associated to transportation;
- Demand satisfaction (C7): Maximizing the satisfaction of customer demands amounts to maximizing the quality of the transportation service, which is influenced by improving the timeliness of transportation process [64] and maximizing the safety of the transported products;
- Flexibility (C8): The use of the milk run increases the flexibility;
- Regulatory criteria (C9): Each activity in the transportation process should be carried out according to requirements related to the specificities of transported products.

A summary of some reviewed studies on the design of the appropriate transportation-network option, considering the different criteria that have been already discussed, is shown in Table 3.

### 3.4. Stakeholder-Engagement Management

Stakeholder management is regarded as an effective approach to manage conflicting stakeholder interests and to improve the quality of decisions to be made by taking into account the diverse interests of all legitimate stakeholders [74,75].

**Figure 4:**
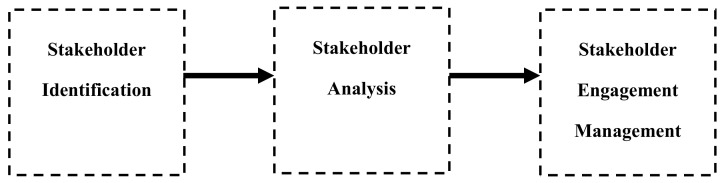
Stakeholder-management processes adopted from [76].

The procedures involved in stakeholder-management process have been widely discussed in existing literature. Summarizing these procedures, we have considered in our previous work [76], the stakeholder identification, stakeholder analyze, and stakeholder engagement as the three essential stages in the stakeholder-management process for a decision-making process (See Figure 4).

As depicted in Figure 4, the first process for managing the stakeholders involves identifying all persons, groups, or organizations that may affect or be affected by the decision to be made. The second process is to analyze the qualitative and quantitative information of the identified stakeholders to determine the key ones to be fully engaged in making appropriate decisions. The third process is the management of stakeholder engagement; it concerns the communication, involving and improving relationships between the stakeholders, with the aim of satisfying their respective needs and expectations.

Several researchers have highlighted the importance of managing the stakeholder participation for improving the quality of the transportation decision-making process. We have provided, in our previous work [76], a summary of various studies on stakeholder management in transportation decision-making process. For example, Batheram et al. [77] considered the management of public participation as prerequisite for the success of the transportation planning. In addition, Wei et al. [78] noted that managing the diverse interests and concerns of stakeholders is considered as a crucial factor for achieving successful transportation decisions. In addition, Cascetta et al. [8] highlighted the role of managing the involvement of stakeholders in bringing about better transportation decisions.

### 3.5. Product-Portfolio Analysis

Another factor that often is left out of discussions on transportation decision making is looking at the actual physical characteristics of what is being shipped, such as type of product, weight, value, perishability, handling characteristics, and packaging [79,80]. Several researchers have highlighted the importance of taking into consideration the products characteristics in making transportation decisions. Rushton et al. [81] noted that considering the physical characteristics and needs of a product is the primary factor that should be taken into consideration in selecting the appropriate transportation mode. In addition, Jeffs et al. [82] considered the product characteristics as one of the important parameters that should be taken into account in determining the appropriate transportation model. In addition, reference [83] highlighted the importance of taking into account the product characteristics in designing the appropriate transportation-network structure. Moreover, reference [84] highlighted the importance of taking into account the characteristics of fresh agricultural products in developing a green transportation system. Hence, it is very important to take into account the characteristics of the transported product in choosing the appropriate transportation.

## 4. Application of the Proposed Framework: Case of the Moroccan Public Pharmaceutical Supply Chain

In this section, we apply our suggested approach to design a transportation strategy for the public pharmaceutical products supply chain in Morocco. In the first stage, we identify the context of the study, and we define the transportation strategy’s objectives that must be achieved. In the second stage, we analyze the actual transportation strategy to help create a clear understanding of what needs to be decided. After that, we identify all the stakeholders that may affect or be affected by the design of the Moroccan public pharmaceutical transportation strategy. Finally, we conduct a decision-making process regarding the design of the transportation network, while involving the relevant stakeholders and taking into account the specificities of pharmaceutical products, in order to design a successful transportation strategy.

### 4.1. Step 1: Context and Problem Definition

In recent years, Morocco launched a number of actions and strategies to enhance access to health services and improve health outcomes for the whole population. In this context, the Moroccan ministry of health had begun to establish a series of actions to improve the level of accessibility and availability of pharmaceutical products for Moroccan citizens.

With the generalization of basic health coverage in 2012, public hospitals must meet the increasingly important demand for care and must face a population that has become more demanding. To adapt to this strong demand, the annual budget allocated to the acquisition of pharmaceuticals has dramatically increased from 300 million dirhams in 2003 to more than 2 billion dirhams in 2018 [85].

Faced with this situation, the current logistic circuit seems to be saturated and no longer adapted to the new challenges. For this reason, the Moroccan Ministry of Health expressed the need to develop an effective and efficient pharmaceutical supply chain through the proposition of strategic and tactical decisions with respect to the following six dimensions: (1) distribution networks, (2) facilities and installations, (3) inventory management, (4) transportation, (5) outsourcing (public–private partnership), and (6) the new technologies and information system [81].

**Figure 5:**
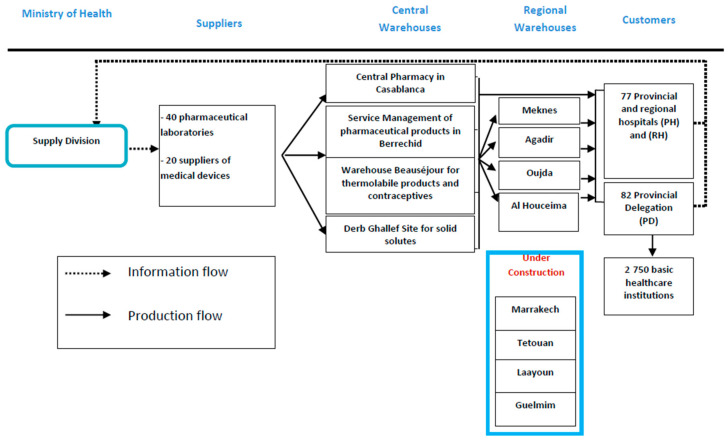
Moroccan public pharmaceutical supply chain.

As can be seen from Figure 5, the Moroccan public pharmaceutical supply chain is organized around several actors [85]:

- Supply division: Responsible for the consolidation of requirements of the different health institutions, the tenders’ launch, the reception, the storage, the distribution of pharmaceutical products, and the administrative management, as well as the supply monitoring.
- Pharmaceuticals suppliers: 40 pharmaceutical laboratories and 20 suppliers of medical devices.
- Central warehouses: which are four in number (Central Pharmacy in Casablanca, service management of pharmaceutical products in Berrechid, Beauséjour warehouse for thermolabile products and contraceptives, and Derb Ghallef Site for solid solutes).
- Regional warehouses: They include four completed (Meknes, Agadir, Oujda and Al Hoceima) and four under construction (Tetouan, Marrakech, Guelmim, and Laayoune).
- Public health institutions (customer zones): which are 159 in number, including 77 provincial and regional hospitals (PH) and (RH) and 82 provincial delegations (PD), which is in charge of supplying 2750 basic healthcare institutions.

Pharmaceutical products should be available in public health institutions at all times in sufficient quantities, delivered and administered safely. In this context, Morocco is making a major effort at increasing the budgets allocated to the acquisition of pharmaceutical products and developing techniques and measures providing health institutions the means to participate in defining their needs [85]. Despite these efforts, several problems persist which are particularly related to the lack of responsiveness of the global chain, the lack of traceability of the logistic circuit of pharmaceutical products, which increased the risk of damage, and the unavailability of pharmaceuticals, which seriously damage the quality of care and causes patient dissatisfaction.

To address these problems, different decisions should be made in relation to the different flows of goods between suppliers and customers including facility location, production, inventory, and transportation [1,63].

Transportation is one of the major supply-chain drivers, as it is responsible for linking the whole supply chain from suppliers to customers. In this sense, we are going to design an appropriate transportation strategy that improves the efficiency and responsiveness of the Moroccan public pharmaceutical supply chain.

### 4.2. Step2: Transportation Strategy Analysis

Transportation of pharmaceutical products is the most important part in a logistics chain because the products being transported are for human consumption. Therefore, it is necessary to develop an appropriate transportation system to ensure that the medicines arrive to consumer in perfect conditions.

In the Moroccan public sector, the deliveries of pharmaceuticals are made by the supply division of the Ministry of Health in a planned way with typically four deliveries per year with an annual volume of 25,000 tons of products.

The products are transported by using small trucks or small vans from suppliers to the storage sites of the Moroccan Ministry of Health, which are divided into central and regional warehouses. The central warehouse usually has copious storage capacity, and it is in charge in delivering the received products, either directly or via regional warehouses, to the customer zones, including provincial and regional hospitals (PH and RH) and provincial delegations (PD) by using smaller vans and cars.

The distance for the transportation of pharmaceutical products is about 700,000 km yearly. The supply division is responsible for delivering pharmaceuticals to provincial and hospital pharmacies through contractual transportation companies. Meanwhile, basic healthcare facilities are delivered by the delegations’ own transportation means. The trucks used for transporting products have different capacities depending on the demand to be delivered (19 tons, 25 tons, and 7 tons).

The Moroccan pharmaceutical-products transportation system knows the intervention of a multiplicity of stakeholders with different levels of competences and interests. As depicted in Table 4, we elaborated on a list of the stakeholders interested in designing the pharmaceuticals transportation system, according to information from our meetings with some professionals of the Moroccan Ministry of Health, our visits to some pharmaceuticals warehouses, such as “the management of pharmaceutical products service in Berrechid”, and based on the literature review about the healthcare and transportation sectors.

### 4.3. Step 3: Group Decision-Making Process

Designing the appropriate pharmaceuticals transportation strategy involves various groups of stakeholders with different interests and wants, which makes achieving their satisfaction a difficult objective to realize. In this sense, it is of great importance to prioritize them to retain the most critical ones to be fully engaged in the design process in order to satisfy their needs and expectations.

#### 4.3.1. Analyze the Identified Stakeholders

We have used the DELPHI method [86] to complete the established list of stakeholders and to prioritize them according to their degree of “having an influence on” and “being impacted by” the design of the transportation strategy of the pharmaceutical products in the Moroccan public sector. A key advantage of the Delphi method is its openness to a diverse set of expert opinions without having to bring everyone together for a physical meeting [87]. Also, it is considered to be an effective method for gaining judgments on complex matters where precise information is unavailable [87]. In addition, the DELPHI method has been widely used to determine consensus in a number of important need areas, especially in the healthcare field [88,89,90].

We have been inspired by the work of [90] in applying the following steps of the Delphi method:

Identification of panel of experts: They should have relevant knowledge and experience in their respective fields. In this manner, 16 people were identified and contacted either directly or via email. Through feedback, 10 of them expressed their agreement to be part of the panel as shown in Table 5.

Design the questionnaire: Firstly, we demanded to each expert to consult the established list of stakeholders and to add others that may be considered pertinent. Then, we outlined some dimensions regarding the transportation of pharmaceuticals to be considered in the questionnaire, so the experts could have a common understanding of the study context (Table 6). After that, we demanded that each expert assign two scores to each stakeholder regarding their “having an influence on” and “being impacted by” the different dimensions concerning the design of the transportation strategy of pharmaceuticals, using a scale from 1 to 5: very low (1), low (2), medium (3), high (4), and very high (5).

Consultation of the experts and analysis of results: The questionnaire has been administered to each expert by email or via direct contact. Then, the questionnaires are returned to be analyzed, aggregated, and shared with the different experts. Several rounds of questionnaires can be sent out to the group of experts until arriving at a group consensus. The degree of consensus among experts is assessed using Kendall’s W coefficient of concordance. This coefficient measures the extent to which the experts agree on their rankings, it ranges between 0 (Perfect disagreement) and 1 (perfect agreement). For a value of W less than 0.7, the questionnaire should be sent back to experts [91]. All experts found that the established list containing all the stakeholders that should be involved in designing the appropriate transportation strategy for the Moroccan public pharmaceutical sector.

To analyze the experts’ answers, we started by calculating the sum of the scores assigned to each stakeholder regarding their “having an influence on” and “being impacted by” the different mentioned dimensions. Then, the completed experts’ responses were analyzed using IBM SPSS Statistics software (IBM, Armonk, NY, USA). The first Delphi round indicated values of W less than 0.7. Hence, the questionnaire was sent back a second and third time to each expert for new responses, accompanied by his or her own rating in the previous rounds and the consolidated result of the panel’s responses. The third round showed an increase of W, which exceeded 0.7, indicating a better degree of consensus (Table 7).

**Figure 6:**
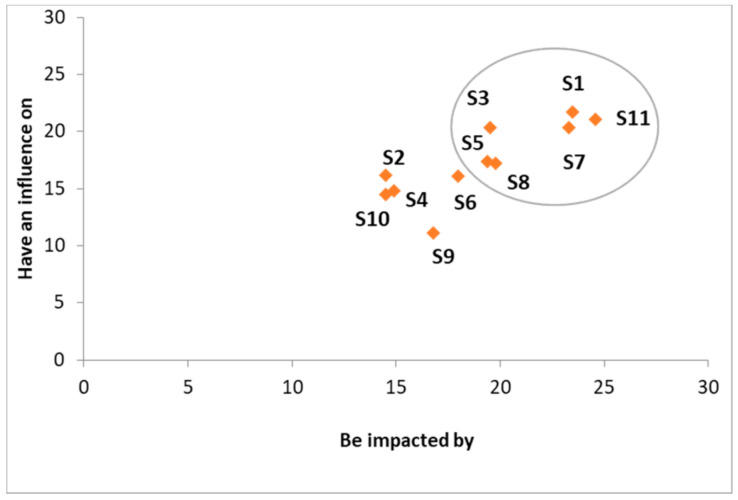
Influence/impact matrix regarding the identified stakeholders. S1: Stakeholder 1; S2: Stakeholder 2; S3: Stakeholder 3; S4: Stakeholder 4; S5: Stakeholder 5; S6: Stakeholder 6; S7: Stakeholder 7; S8: Stakeholder 8; S9: Stakeholder 9; S10: Stakeholder 10; S11: Stakeholder 11.

For a better understanding of this prioritization, we have put the stakeholders in the influence/impact matrix proposed by [92] (Figure 6).

As can be seen from Figure 6, all the identified stakeholders are both influencing and influenced by designing an appropriate transportation strategy for the Moroccan pharmaceutical public sector. Nevertheless, six stakeholders have the highest score: patients (S11), Ministry of Health (S1), public healthcare facilities and healthcare personal (S7), Ministry of Transport (S3), healthcare industry (S8), and local authorities (S5). Those stakeholders are the most critical ones with regard to the design of the appropriate pharmaceuticals transportation strategy, and they need to be fully engaged in the decision-making process in order to satisfy their needs and expectations. However, the other stakeholders should be adequately informed in order to keep their satisfaction and to maintain their engagement.

However, it should be noted that the identified stakeholders and their obtained classification can be updated at regular intervals over time, according to the happened changes in the environment.

Moreover, designing the appropriate pharmaceuticals transportation strategy requires decision making regarding different components: designing the appropriate transportation network, choosing the suitable transportation mode, and deciding which part of the transportation process will be outsourced and which one will be insourced, while involving the relevant stakeholders and taking into account the specificities of the pharmaceutical products. In this paper, we are interested in designing the transportation network.

In the following section, we conduct a decision-making process to choose the most appropriate transportation-network design option for the Moroccan public pharmaceutical supply chain while involving the main stakeholders and taking into account the specificities of pharmaceutical products.

#### 4.3.2. Choosing the Appropriate Transportation Network

We have proposed, in a previous work [63], a framework for designing the appropriate transportation network for a given supply chain, and we have illustrated the application of this framework in choosing the most appropriate transportation-network design option for the Moroccan public pharmaceutical supply chain.

However, we realized that the analysis of the transportation system should involve many social groups and different aspects. Accordingly, we have decided to engage the identified key stakeholders in choosing the appropriate transportation-network design option for the public pharmaceutical supply chain in Morocco.

Four transportation-network design options were proposed in [63]:

✓ (TN1): Products shipping via central/regional warehouses;
✓ (TN2): Products shipping via central/regional warehouses by using milk run from suppliers to central warehouses;
✓ (TN3): Products shipping via central/regional warehouses by using milk run from regional warehouses to a supply chain’s customers;
✓ (TN4): Products shipping via central/regional warehouses by using milk run from suppliers to central warehouses and from regional warehouses to a supply chain’s customers.

The choice of the appropriate transportation-network design option is based on a set of criteria (Cj). Based on the previous list of criteria determined according to the literature (Table 3), six criteria that could glue to our context have been selected.

Criteria to minimize:

- Delivery time: the actual time between placing an order and receiving the delivered product.
- Number of trucks: the use of milk run reduces the number of trucks utilized for shipping pharmaceutical products.
- Transportation cost: consolidating shipments lowers transportation cost.
- Information system cost: the use of milk runs increases the cost of information system because it requires a significant degree of coordination.
- Criteria to maximize:
- Regulatory criteria: Transporting pharmaceutical products should be carried out according to requirements of the drugs act in order to avoid the risk of impacting the safety, quality, and effectiveness of pharmaceuticals.
- Flexibility: Ability to respond to unexpected events in order to satisfy the customer’s specific needs.

Six key stakeholders were contacted representing the six main interest stakeholder groups which have already been mentioned. Through feedback, only five stakeholders expressed their agreement to participate in the decision-making process: (1) Ministry of Health (S1), (2) Ministry of Transport (S3), (3) public healthcare facilities and healthcare personnel (S7), (4) healthcare industry (S8), and (5) patients (S11). Stakeholders were individually contacted and approached directly or through emails explaining the objective of the study with a detailed description of the four different alternatives in terms of strengths and weaknesses. Then, they were asked to confirm the established list of criteria. Nevertheless, deciding io the appropriate transportation network while engaging the key stakeholders and taking into account the different criteria needs to be tackled with a multicriteria decision-making approach.

Many MCDA methods exist, but the analytic hierarchy process (AHP), proposed by [93], is used in this paper because of its simplicity, its flexibility, and its ability to deal with different types of information features [94]. Furthermore, throughout this method, the hierarchy is revealed after the breakdown of the problem, which enables understanding and defining the process itself [95]. The AHP method has been widely used to support decision making in several and different fields, especially in transportation issues [7,12,96,97].

In addition, as different persons affect the decision-making process, the AHP method has been adapted to be applied in group decision making. Hence, it is considered a useful method to support stakeholder engagement in making appropriate decisions [14,98].

There are different ways to combine the preferences of the decision makers into a consensus rating [12]: according to the level of aggregation (from judgments or from priorities) and the type of aggregation (mathematical or based on consensus vote). The consensus vote is used when stakeholders reach an agreement on the values of the matrices or on the priority vectors; otherwise, the mathematical aggregation is adopted. There are two synthesizing methods that provide the same results in case of acceptable consistency of the pairwise comparison matrices [12]: the weighted geometric mean method (WGMM) or weighted arithmetic mean method (WAMM). In the first method, the geometric means of individual judgements are used as elements in the pairwise comparison matrices and then priorities are computed. Meanwhile, in the second method, priorities are computed and then aggregated using the weighted arithmetic mean method [12].

In the following section, we applied the different steps of the AHP method [93] to select the most appropriate transportation network for the Moroccan public pharmaceutical supply chain.

**Figure 7:**
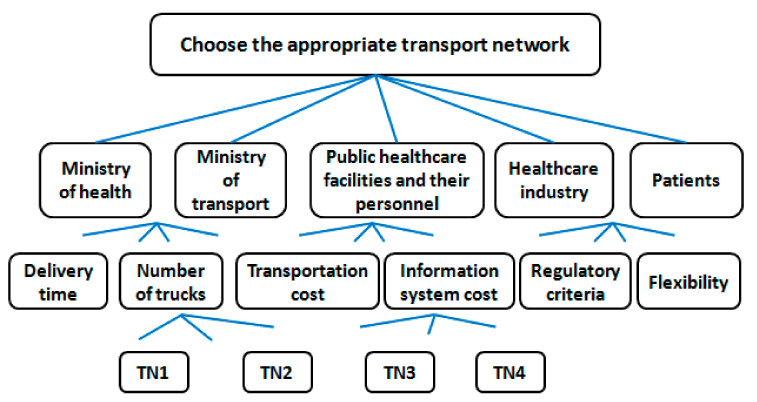
Hierarchy of decision. TNi: Transport network design options. (TN1): Products shipping via central/regional warehouses; (TN2): Products shipping via central/regional warehouses by using milk run from suppliers to central warehouses; (TN3): Products shipping via central/regional warehouses by using milk run from regional warehouses to a supply chain’s customers; (TN4): Products shipping via central/regional warehouses by using milk run from suppliers to central warehouses and from regional warehouses to a supply chain’s customers.

First, a hierarchical structure of the problem was modeled (Figure 7). Starting with the top level, the global goal is to choose the most appropriate transportation network. The second level identifies the main stakeholders that are involved in the decision making. In the third level, we present the evaluation criteria. Finally in the last level, the different transportation-network alternatives (TNi) are presented, from which we will be able to choose the best transportation-network option. After a brief reminder of the AHP methodology to the different key stakeholders, they were given the opportunity to revise the hierarchy model and to validate it.

Then, the stakeholders’ weight was evaluated from the stakeholders themselves (self-assessed weights) through a questionnaire. The judgements were justified by the analysis of the results of DELPHI method (Table 8).

**Figure 8:**
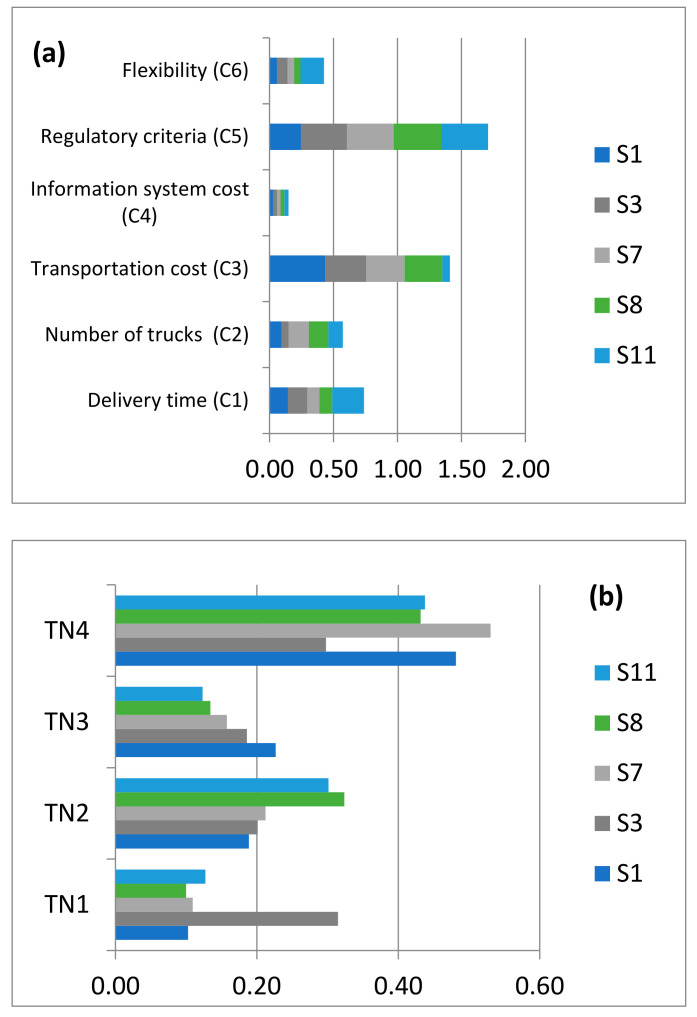
Priority results of (**a**) criteria and (**b**) alternatives. S1: Stakeholder 1; S3: Stakeholder 3; S7: Stakeholder 7; S8: Stakeholder 8; S11: Stakeholder11. TNi: Transport network design options. (TN1): Products shipping via central/regional warehouses; (TN2): Products shipping via central/regional warehouses by using milk run from suppliers to central warehouses; (TN3): Products shipping via central/regional warehouses by using milk run from regional warehouses to a supply chain’s customers; (TN4): Products shipping via central/regional warehouses by using milk run from suppliers to central warehouses and from regional warehouses to a supply chain’s customers.

After that, each of the stakeholders was asked to make pairwise comparisons for the different alternatives against each criterion and for the different criteria while verifying the consistency. Then, an aggregation of the priority vectors of different alternatives, while taking into account the criteria weights, was performed in order to obtain the ranking of alternatives according to each stakeholder (Figure 8b).

As can be seen from Figure 8a, the regulatory criteria are considered to be the most important criterion, followed by transportation cost, delivery time, number of trucks, flexibility, and information system cost. These results are clearly in line with the preferences that key stakeholders expressed in questionnaires. Regarding the priority vector of alternatives as shown in Figure 8b, the fourth transportation-network alternative (TN4) shows the highest priority for four stakeholders (S1, S7, S8, and S11), while the first transportation-network alternative (TN1) is the first ranked for the Ministry of Transport (S3).

**Figure 9:**
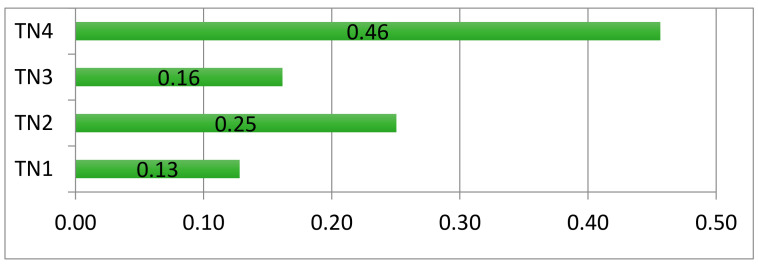
Aggregation of transportation-network-alternatives ranking. TNi: Transport network design options. (TN1): Products shipping via central/regional warehouses; (TN2): Products shipping via central/regional warehouses by using milk run from suppliers to central warehouses; (TN3): Products shipping via central/regional warehouses by using milk run from regional warehouses to a supply chain’s customers; (TN4): Products shipping via central/regional warehouses by using milk run from suppliers to central warehouses and from regional warehouses to a supply chain’s customers.

In the following section, aggregation of individual priorities has been performed, while applying the weighted arithmetic mean method to derive a collective decision as an output of the AHP methodology (Figure 9). A consultation process with the five stakeholders was structured through conducting interviews over the telephone and face-to-face meetings, in order to show them the final results that confirm that the TN4 with the highest score of 0.46 is the most appropriate transportation network for the current Moroccan public pharmaceutical distribution network. We have also discussed the benefits of the chosen option which are decreasing transportation cost through consolidation and reducing the number of trucks utilized for shipping pharmaceuticals through the use of milk run. Also, using milk run reduces the inventory-storage level, preventing the pharmaceuticals from being stored for a long time, which can impact their safety, quality, and effectiveness. 

## 5. Discussion

Transportation is at the heart of supply-chain operations, as it is the responsible for ensuring the movement of products from supply sites to customers. Deciding on the appropriate transportation strategy requires the participation of multiple stakeholders with different interests and expectations. The main objective of this paper was to propose a framework for designing an appropriate transportation strategy for a supply chain from a multistakeholder perspective. This research work was born from the need of the Moroccan Ministry of Health in improving the efficiency and the responsiveness of the pharmaceutical public supply chain in order to satisfy the increasing demand of pharmaceuticals. We have applied the suggested framework for deciding on the appropriate strategy for transporting pharmaceuticals in the Moroccan public sector.

First, an analysis of the current pharmaceuticals supply chain was carried out in order to have a clear understanding of the actual situation, to determine the multiple daunting challenges faced by the current supply system, and to define the objectives that must be achieved to meet the Ministry of Health‘s expectations. Then, we analyzed the current transportation strategy regarding its three components: transportation network, transportation mode, and transportation outsource/insource. We established a list of the different stakeholders interested in the design of the pharmaceuticals transportation strategy for the Moroccan public sector. The identification of stakeholders was done based on the literature review conducted on previous studies on healthcare and transportation sectors, and also, through the analysis of some conducted interviews and work meetings with the professionals of the Ministry of Health. These stakeholders were prioritized, while applying the DELPHI method, according to their degree of “having an influence on” and “being impacted by” the design of the transportation strategy of the pharmaceutical products in the Moroccan public sector. The findings have allowed the identification of six key stakeholders to be fully engaged in transportation strategy decision-making process:

- Patients (S11): They are at the core of the health system. The World Health Organization emphasized to put the patients at the center of the healthcare system by meeting their needs and expectation [99]. The patients are the most impacted by the design of the appropriate pharmaceuticals transportation strategy by providing them high-quality pharmaceuticals whenever and wherever they are needed.
- Ministry of health (S1): It plays a crucial role in the design of the pharmaceuticals transportation strategy through strengthening the Moroccan’s healthcare system and having the overall responsibility for the management and development of that system.
- Public healthcare facilities (S7): They are the primary means of the healthcare delivery to patients. They play a potential role in the design of the pharmaceuticals transportation strategy through their location in the distribution network, their respect of patient’s preferences, and their ability to ensure the availability of pharmaceutical products to patients.
- Ministry of Transport (S3): It plays an important role in the design of the pharmaceutical transportation strategy, as it is responsible for transportation within the Moroccan’s country including overseeing road safety and developing government transportation policies.
- Healthcare industry (S8): It plays a key role in the healthcare system as it is the responsible of the production and the distribution of healthcare products, especially the pharmaceuticals, to the different healthcare institutions.
- Local authorities (S5): They represent a key participant in the implementation of national healthcare strategies.

We contacted six stakeholders representing the identified key stakeholder groups for participating in the transportation strategy decision-making process. Through feedback, only five stakeholders expressed their agreement to participate. (1) Ministry of Health (S1), (2) Ministry of Transport (S3), (3) public healthcare facilities and healthcare personnel (S7), (4) healthcare industry (S8), and (5) patients (S11).

After that, we applied the AHP Group method to choose the appropriate transportation-network design option for the Moroccan public pharmaceutical supply chain while involving the identified key stakeholders into. The results showed that applying the milk-run option from suppliers to central warehouse, as well as from regional warehouses to customer zones, is the most suitable transportation-network option for shipping the pharmaceutical products in the Moroccan‘s current public distribution network.

The findings of this research work raise some interesting research questions and create promising perspectives for future work. We summarize below a list of some potential research venues:

- Deciding on the others transportation strategy’s components while involving the relevant stakeholders into choosing the appropriate transportation mode, as well as deciding which part of the transportation process will be outsourced and which one will be insourced.
- The proposed transportation strategy decision-making process was conducted while engaging five key stakeholders that represent five key stakeholder groups: namely, (1) Ministry of Health, (2) Ministry of Transport, (3) public healthcare facilities and healthcare personnel, (4) healthcare industry, and (5) patients. However, in order to improve the quality of the decisions to be made, we look for involving others performing persons representing the key stakeholder groups.
- Having a clear insight on the stakeholders involved in the decision-making process and the interactions among them is helpful in guiding an effective participation process aimed at consensus building among stakeholders. In this sense, we propose, for our future work, to use a modeling approach based on participatory modeling methods such as: the social network analysis (SNA), discrete choice models (DCM), and agent-based models (ABM).
- Improving the proposed framework to be adapted for designing a smart transportation strategy through the employment of the digital and telecommunication technologies like information and communications technology (ICT) and the Internet of things (IoTs). The design of such transportation system will have numerous benefits including improving the life quality of the entire population and maximizing the sustainability and environmental conservation [100].
- Another issue that deserved investigating is to use other multi-criteria decision analysis (MCDA) methods adapted to be applied in the group decision-making context participation to evaluate and analyze the performance of the decisions to be made.

## 6. Conclusions

In this paper, we presented a framework for designing an appropriate transportation strategy for a supply chain while involving the concerned stakeholders and taking into account the specificities of the transported products.

This framework was applied to a real case study of the Moroccan public pharmaceutical supply chain. First, we defined the transportation strategy decision-making context and the objectives that must be achieved. Second, we analyzed the current transportation strategy regarding their three components: transportation network, transportation mode, and transportation insource/outsource. Then, we identified the stakeholders interested in designing the pharmaceuticals transportation system. Finally, in the third step, we conducted a group decision-making process regarding the transportation-network design component. In this sense, the identified stakeholders were first analyzed and hierarchized while employing the DELPHI method to determine the key of them. After that, we have applied the Group AHP MCDA method for selecting the appropriate transportation-network design option while involving the identified key stakeholders into. The results showed that shipping products via central/regional warehouses with using milk run from suppliers to central warehouses and from regional warehouses to a supply chain’s customers is the most suitable transportation-network option for shipping the pharmaceuticals in the Moroccan’s public pharmaceutical supply chain.

Future work will deal with choosing the appropriate transportation mode, as well as deciding which part of the transportation process will be outsourced and which one will be insourced, while involving the relevant stakeholders.

## Figures and Tables

**Table 1:** A summary of criteria used in literature to select the appropriate transportation mode.

Ref.	Criteria						
	C1	C2	C3	C4	C5	C6	C7
[1]		x	x	x	x		
[28]		x	x	x	x	x	x
[30]	x	x	x	x	x		
[33]	x	x		x			
[34]				x	x		
[35]		x	x		x		
[36]		x			x		
[29]		x			x		
[37]		x			x		
[38]			x	x	x		
[39]		x		x	x		
[40]		x	x	x	x		
[41]		x	x	x	x	x	x
[42]	x			x	x		
[43]	x				x		

Ref.: References; C1: Customers/suppliers density and distance; C2: Shipment size; C3: Product characteristics; C4: Delivery time; C5: Total cost; C6: Flexibility; and C7: Safety criteria.

**Table 2:** A summary of criteria used in literature to decide on transportation insourcing/outsourcing.

Ref.	Criteria											
	C1	C2	C3	C4	C5	C6	C7	C8	C9	C10	C11	C12
[1]												x
[44]	x		x	x			x					
[46]	x	x		x					x		x	
[47]	x	x		x								
[48]			x									
[49]		x	x									
[50]	x	x										
[51]	x	x										
[52]	x	x						x			x	
[53]	x	x									x	
[54]	x								x			
[55]	x			x				x	x			
[56]	x	x	x	x	x	x	x	x		x	x	
[57]	x	x	x	x	x	x	x	x	x	x	x	
[58]	x	x	x							x		
[59]	x					x						
[60]	x	x										
[61]	x	x		x								
[62]	x						x					

Ref.: References; C1: Total cost; C2: Core competency; C3: Technological capability; C4: Flexibility; C5: Responsiveness; C6: Quality; C7: Timeliness; C8: Information security; C9: Quality Control; C10: Product complexity and specificity; C11: Risks; and C12: Shipment size.

**Table 3:** A summary of criteria used in literature in designing transportation network.

Ref.	Criteria								
	C1	C2	C3	C4	C5	C6	C7	C8	C9
[63]	x	x	x	x					x
[65]	x		x			x			
[66]					x	x			
[67]					x		x		
[68]	x								
[69]	x						x		
[70]	x		x			x	x	x	
[71]	x		x			x			
[72]		x					x		
[73]	x					x			

Ref.: References; C1: Delivery time; C2: Number of trucks; C3: Transportation cost; C4: Information system cost; C5: Size of served area; C6: Transportation risk; C7: Demand satisfaction; C8: Flexibility; and C9: Regulatory criteria.

**Table 4:** List of the identified stakeholders.

Number	Stakeholders	Interests
S1	Ministry of Health	Leads the Moroccan’s healthcare system and has overall responsibility for the management and development of that system
S2	Ministry of Economy and Finance	Support the development and improvement of the healthcare system by defining the budget allocated to the implementation of health programs
S3	Ministry of Transport	Responsible of road network development and transportation policies
S4	Ministry of Environment	Responsible for the government’s environmental program while taking all appropriate measures for the protection of the environment
S5	Local authorities	Participate in implementation of national strategies in different fields and particularly in the health sector
S6	International health organizations (World Health Organization—(WHO), etc.)	Support national health policies and strategies and provide norms and standards that should be respected in designing healthcare strategies
S7	Public healthcare facilities and healthcare personnel	Provide essential healthcare services to patients
S8	Healthcare industry: Suppliers, wholesalers, and distributors	Responsible for the development, production, and distribution of pharmaceutical products to the different healthcare facilities
S9	National institutions and associations: Mohammed VI foundation for the environmental protection, Economic, Social and Environmental Council, etc.	Provide advisory services to the government on social, human, and environmental policies.
S10	Media	Promote healthcare information to the public
S11	Patients	Benefit from the pharmaceutical products as they are the final customers of the pharmaceutical supply chain

S1: Stakeholder 1; S2: Stakeholder 2; S3: Stakeholder 3; S4: Stakeholder 4; S5: Stakeholder 5; S6: Stakeholder 6; S7: Stakeholder 7; S8: Stakeholder 8; S9: Stakeholder 9; S10: Stakeholder 10; S11: Stakeholder 11.

**Table 5:** List of experts involved in the DEPLHI survey.

Number	Affiliation	Degree	City
1	University Hospital	Anesthesia nurse	Casablanca
2	Prefectural Hospital	Nurse	Casablanca
3	Prefectural Hospital	Nurse	Casablanca
4	University Hospital	Doctor	Casablanca
5	Provincial Hospital	Doctor	El Jadida
6	Ministry of Health	Engineer	Rabat
7	Ministry of Health	Doctor	Rabat
8	Ministry of Equipment, Transport, and Logistics	Engineer	Rabat
9	Ministry of Economy and Finance	Engineer	Rabat
10	University	Healthcare researcher	Rabat

**Table 6:** Dimensions regarding the transportation of pharmaceuticals used in the questionnaire.

Dimensions	Definition
Effectiveness	An effective pharmaceuticals transportation system leads to better outcomes and more satisfied patients in terms of transportation time saving
Efficiency	The transportation of pharmaceuticals should be provided with the appropriate use of resources (Minimum level of fleets used and minimum transportation cost)
Security	The transportation of pharmaceutical products must be carried following the good practices defined by the Ministry of Health and the regulations in force to ensure that the products arrive in good quality to the patients.
Availability	Ensure the availability of pharmaceutical products to the patient at any time.
Equity	Guarantee the availability of pharmaceutical products to the entire population without discrimination.

**Table 7:** Results of Delphi survey of stakeholder prioritization.

Stakeholders	Have an Influence on						Be Impacted by					
	First Round		Second Round		Third Round		First Round		Second Round		Third Round	
	Mean (In_k_)	Standard Deviation	Mean (In_k_)	Standard Deviation	Mean (In_k_)	Standard Deviation	Mean (Im_k_)	Standard Deviation	Mean (Im_k_)	Standard Deviation	Mean (Im_k_)	Standard Deviation
S1	22,20	4.077	20.90	1.449	21.70	3.057	19.60	2.757	19.70	2.710	23.50	2.593
S2	19.80	4.158	16.70	1.494	16.20	1.619	14.10	1.912	14.10	1.912	14.50	2.014
S3	21.60	3.718	20.40	3.534	20.30	2.312	14.90	2.846	15.30	2.111	19.50	2.759
S4	15.60	4.033	13.60	2.366	14.80	1.687	13.10	3.665	13.70	2.497	14.90	1.969
S5	20.10	2.961	17.10	1.370	17.40	1.647	17.50	2.799	17.50	2.799	19.40	2.066
S6	17.70	3.683	16.40	2.547	16.10	1.663	15.40	2.836	15.80	2.741	18.00	2.944
S7	21.00	2.749	21.50	2.173	20.30	3.093	21.70	1.947	21.70	1.947	23.30	2.003
S8	21.10	3.784	17.80	2.700	17.20	2.150	16.80	2.974	16.80	2.974	19.80	3.994
S9	13.00	3.367	12.90	2.079	11.10	1.729	15.10	4.280	15.60	3.098	16.80	2.741
S10	15.70	2.627	15.00	2.000	14.50	1.958	13.30	3.199	13.90	1.663	14.50	1.269
S11	14.90	3.573	19.20	1.874	21.10	0.876	24.60	0.516	24.60	0.516	24.60	0.516
Kendall’s W coefficient of concordance	0.643		0.690		0.810		0.684		0.687		0.820	

S1: Stakeholder 1; S2: Stakeholder 2; S3: Stakeholder 3; S4: Stakeholder 4; S5: Stakeholder 5; S6: Stakeholder 6; S7: Stakeholder 7; S8: Stakeholder 8; S9: Stakeholder 9; S10: Stakeholder 10; S11: Stakeholder 11.

**Table 8:** Pairwise comparison of main stakeholders.

Stakeholders	Ministry of Health (S1)	Ministry of Transport (S3)	Public Healthcare Facilities and Their Personnel (S7)	Healthcare Industry (S8)	Patient (S11)	Relative Importance
Ministry of Health (S1)	1	5	3	7	1/3	0.27
Ministry of transport (S3)	1/5	1	1/5	3	1/6	0.06
Public healthcare Facilities and their personnel (S7)	1/3	5	1	7	¼	0.17
Healthcare industry (S8)	1/7	1/3	1/7	1	1/7	0.03
Patients (S11)	3	6	4	7	1	0.47

For *n* = 5, λmax = 5.44, CI (Consistency Index) = 0.11, CR (Consistency Ratio) = 0.09 < 0.1 OK.

## Data Availability

The data presented in this study are available on request from the corresponding author.
